# Comparison of Normothermic and Subnormothermic Machine Perfusion of Porcine Kidneys Using a Novel Fully Synthetic Perfusion Solution: A Proof-of-Concept Study

**DOI:** 10.3390/jcm15062287

**Published:** 2026-03-17

**Authors:** Hannah K. Krüger, Benedict M. Doorschodt, Zoltan Czigany, Oliver Beetz, Felix Oldhafer, Alexander Theißen, Laura Zarnitz, Lisa Ernst, Felix von Lendenfeld, Jan Larmann, René H. Tolba, Christian Bleilevens

**Affiliations:** 1 Department of Anesthesiology, Medical Faculty, RWTH Aachen University Hospital, 52074 Aachen, Germany; atheissen@ukaachen.de (A.T.); lauzarnitz@ukaachen.de (L.Z.); jlarmann@ukaachen.de (J.L.); 2 Vivalyx GmbH, 51105 Cologne, Germany; 3 Department of Surgery & Transplantation, University Hospital Heidelberg, Medical Faculty, Ruprecht Karl University Heidelberg, 69120 Heidelberg, Germany; zoltan.cziganymd@gmail.com; 4 Department of General, Visceral, Pediatric and Transplant Surgery, Medical Faculty, RWTH Aachen University Hospital, 52074 Aachen, Germany; obeetz@ukaachen.de (O.B.); foldhafer@ukaachen.de (F.O.); 5 Institute for Laboratory Animal Science and Experimental Surgery, Medical Faculty, RWTH Aachen University, 52074 Aachen, Germany; lernst@ukaachen.de (L.E.); rtolba@ukaachen.de (R.H.T.)

**Keywords:** kidney transplantation, organ preservation, kidney machine perfusion, normothermic machine perfusion, subnormothermic machine perfusion

## Abstract

**Background/Objectives**: The growing shortage of organs for transplantation requires optimized preservation techniques. Normothermic (NMP) and Subnormothermic Machine Perfusion (SMP) allow for the assessment of organ viability prior to transplantation and enable targeted therapeutic interventions while maintaining a metabolically active state in contrast to hypothermic settings. **Methods**: In this study, the synthetic perfusion solution “Omnisol” was used in a 6 h ex vivo setting with porcine kidneys (*n* = 6 NMP; *n* = 6 SMP). Perfusion parameters (arterial flow, intrarenal resistance and urinary flow), renal function (excretory and filtration performance), renal injury (cellular and circulating biomarkers) and tissue and perfusate oxygenation were assessed. **Results**: NMP resulted in better arterial flow and lower intrarenal resistance during the first 3 h of perfusion, while SMP surpassed NMP from 3 to 6 h. Renal injury biomarkers increased in the NMP group after 3 h, while no increase was detectable in the SMP group. Omnisol fully met the oxygen requirements of the kidneys in both groups, despite being fully synthetic. **Conclusions**: Both NMP and SMP offer distinct advantages for kidney preservation, and the synthetic perfusate Omnisol appears to be feasible for both methods. In this experimental setting, the data indicate that NMP of porcine kidneys was associated with favorable functional parameters during the early phase of perfusion, whereas SMP showed comparatively stable parameters at later time points. These findings should primarily be considered exploratory observations and require validation in future studies, especially for the translation into a clinical scenario using human kidneys.

## 1. Introduction

Organ shortage remains a demanding challenge in kidney transplantation due to the persisting gap between organ supply and demand [1]. To increase the number of kidneys for transplantation, marginal grafts, including those from donation after circulatory death (DCD) and extended criteria donors (ECDs), are increasingly being utilized [2].

DCD and ECD organs are associated with a higher risk of graft dysfunction or nonfunction, and increased immunogenicity [3,4,5]. Moreover, ischemia–reperfusion injury (IRI) remains a major cause of delayed graft function (DGF) and graft failure, particularly in ECD kidneys [6]. To make optimal use of marginal organs, improved preservation techniques are sought, enabling the assessment of graft function before transplantation.

The clinical standard of organ preservation in most countries remains static cold storage (SCS) [7,8,9]. SCS provides a cost-effective method for kidney preservation and significantly reduces metabolic activity, oxygen demand and thus minimizes cellular damage [7]. However, cold ischemia time inherent to SCS correlates with increased rates of DGF and primary nonfunction (PNF), making marginal grafts especially vulnerable to SCS [10]. Additionally, static cold-stored organs are strongly prone to IRI, as after prolonged ischemia, reactivation of proteinases increases mitochondrial membrane permeability, generating reactive oxygen species and thus triggering oxidative stress, apoptosis and ferroptosis [11].

A more advanced preservation technique is hypothermic oxygenated perfusion (HOPE). HOPE allows for the partial assessment of organ quality and partial ATP production as an indicator of the organ’s energy status and its viability. Moreover, HOPE was shown to be clinically safe and effective [7,12,13], presented better outcomes compared to SCS [14] and is becoming the clinical standard, e.g., in the Netherlands [15], but limitations such as inadequate viability assessment have led to the exploration of normothermic machine perfusion (NMP) [16].

Kidney NMP has shown a significant reduction in preservation injury compared to hypothermic preservation methods [17] and enables organ preservation at near physiological conditions, allowing for the assessment of organ function, making it more suitable for marginal organs [14,18]. Furthermore, NMP enables the resuscitation of marginal grafts [17,19,20] and allows for targeted therapeutic interventions to improve organ quality [21,22].

However, most current NMP protocols rely on complex perfusate compositions including red blood cells (RBCs) as a primary oxygen carrier, human albumin and additional buffer systems [23]. Also, hemolysis resulting in the release of free hemoglobin, which is toxic and can trigger oxidative stress, inflammation, cellular damage and apoptosis [24,25,26,27] is common in ex vivo circuits. Furthermore, the scarcity of RBCs and its cost-intensive nature restrict the widespread adoption of NMP [28]. Acellular nutrient-supplemented perfusates have been proposed as alternatives to RBCs, offering a solution to overcome these challenges [29]. In this context, several synthetic perfusion solutions have been developed and evaluated. The University of Wisconsin (UW) solution, originally developed by Belzer and colleagues [30], remains the gold standard for static cold storage of abdominal organs. The solution has demonstrated excellent preservation efficacy under hypothermic conditions and has been clinically validated over decades. However, its formulation was designed for static cold storage and not for active oxygenated perfusion under normothermic conditions. For warmer perfusion temperatures, solutions can be categorized into hemoglobin-based oxygen carriers (HBOCs) and acellular nutrient-supplemented solutions. HBOCs, such as Hemopure^®^, have been explored as oxygen carriers in ex vivo perfusion models [31], offering advantages such as standardized composition and elimination of hemolysis-related complications, but remaining associated with oxidative stress and vasoconstrictive effects [32]. The acellular synthetic perfusate Steen Solution™, which provides a physiological oncotic pressure as it contains human serum albumin, has been widely used in lung perfusion [33]. These approaches highlight potential advantages of synthetic perfusates, such as improved standardization, reduced immunogenicity, lower infectious risk, no risk of hemolysis and simplified logistics. However, limited clinical validation of newly developed fully acellular perfusion setups compared to blood-based perfusion prevents their widespread clinical implementation.

Another emerging organ preservation method currently being tested preclinically is Sub-Normothermic Machine Perfusion (SMP), combining the advantages of both cold and warm preservation techniques. SMP offers a balance between reduced metabolic demands and sufficient mitochondrial function to replenish ATP [34]. Furthermore, SMP provides the benefits of NMP, such as enabling real-time graft assessment and therapeutic intervention [29,35,36], and was demonstrated to be feasible for the preservation of DCD kidneys [36,37].

Previously, our group successfully performed a 6 h NMP of porcine kidneys using a fully synthetic perfusion solution in comparison to blood-based NMP [38], showing noninferiority of the synthetic solution. The aim of the present study was to evaluate the feasibility of 6 h NMP and SMP of porcine kidneys using a fully synthetic perfusate, including an analysis of perfusion dynamics, metabolic parameters, and markers of cellular injury, hypothesizing that both regimes could be covered using the same perfusate.

## 2. Materials and Methods

### 2.1. Experimental Protocols

The experimental protocol was approved by the governmental State Agency for Nature, Environment and Consumer Protection of North Rhine-Westphalia (LAVE) under license 81-02.04.2022.A402. and all animal experiments were performed in accordance with German legislation governing animal studies following the “Guide for the Care and Use of Laboratory Animals” (NIH publication, 8th edition, 2011) and the Directive 2010/63/EU on the protection of animals used for scientific purposes (Official Journal of the European Union, 2010).

Kidneys (*n* = 6 per group, based on previous work [38,39]) were perfused with Omnisol (Vivalyx GmbH, Aachen, Germany) at either 22.37 ± 0.34 °C (SMP) or at 35.39 ± 0.40 °C (NMP). To minimize inter-animal variability, paired kidneys from the same pig were randomly allocated to different groups (SMP or NMP) and subsequently perfused simultaneously for 6 h. Arterial and venous perfusate and urine samples were collected, and tissue oxygenation was measured at regular time points (0, 5, 30, 60, 120, 180, 240, 300, and 360 min). Core needle biopsies were obtained at time points 0 and 360 min for molecular biology analysis.

All animals used for this study were part of an organ sharing program according to the 3R principle in laboratory animal sciences (Refine, Reduce, and Replace). The remaining organs and tissues of pigs used for this experiment were provided to other in-house institutes and research groups for further experiments.

No animals, kidney or data points were excluded in this study. No criteria for the exclusion of animals or kidneys were predefined.

Due to the technical nature of the surgical setup and the manual operation of the perfusion machine, investigators were aware of the group allocation while conducting the experiment, the outcome assessment, and the data analysis.

### 2.2. Preparation of Kidneys

Six German landrace pigs (body weight: 50.83 ± 1.82 kg) from a hygienically controlled barrier breeding facility (Heinrich Genetics, Heinsberg, Germany) were housed in fully air-conditioned rooms (22 °C room temperature, 50% relative humidity). Animals were acclimatized for at least seven days and were separated into species-appropriate fasting boxes the evening before surgery, with free access to water.

Premedication was performed with an intramuscular (i.m.) injection of 1 mL of 1% atropine (1 mL/1% atropine sulphate, Dr. Franz Köhler Chemie GmbH, Bensheim, Germany) and 2 mL/10 kg body weight (BW) azaperone (Stresnil^®^, Janssen-Cilag GmbH, Neuss, Germany). Additional sedation was provided after 10 min with 1 mL/10 kg BW ketamine (Ceva GmbH, Duesseldorf, Germany) i.m. Propofol (2 mg/kg BW) was administered individually as required for narcosis via an indwelling venous cannula placed in an ear vein. Further details are provided in the Supplementary Materials (Supplementary S1).

Kidneys were explanted simultaneously and washed out with 500 mL of Omnisol, prewarmed to 22 °C or 35 °C and supplemented with heparin (5000 IU/mL, B. Braun Melsungen AG, Melsungen, Germany) via the cannulated renal artery (Retrograde Cardioplegia Catheter, 14 Fr., Edwards Life Sciences, RC014; Unterschleißheim, Germany). Further details are provided in the Supplementary Materials (Supplementary S2).

### 2.3. Subnormothermic and Normothermic Machine Perfusion

After an ex situ ischemic time of 30 min, kidneys were perfused simultaneously at a mean arterial pressure (MAP) of 75 mmHg in both groups. The MAP was gradually increased from 25 to 75 mmHg within the first 15 min of perfusion.

**Figure 1:**
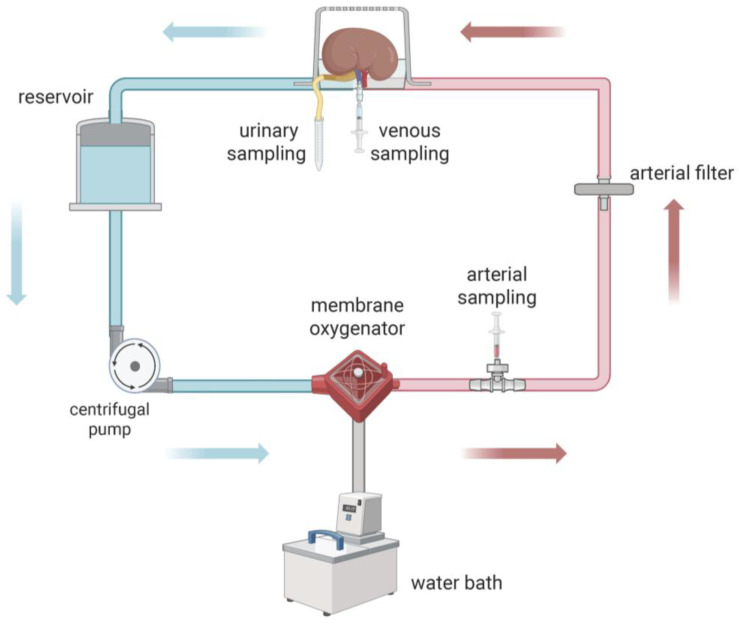
Schematic setup of the perfusion circuit (created with BioRender.com). The setting was identical for both groups, except for the water bath thermostat set to 37 °C in the NMP group or switched off to enable ambient temperature in the SMP group. blue arrows = venous perfusate/red arrows = arterial perfusate.

The perfusion circuit used for this study was previously established by our group [38,39], with the only modification being the use of a custom half-closed wet chamber and a different reservoir (Figure 1). Extracorporeal perfusion circuit hardware was allocated randomly to the experimental group to avoid any bias due to the hardware system. Further details are provided in the Supplementary Materials (Supplementary S3).

Perfusate oxygenation was achieved using an oxygenator (Newborn A.L.ONE ECMO, Eurosets GmbH, Gröbenzell, Germany) connected to a medical oxygen source. The oxygen flow was adjusted individually, based on the arterial perfusate pO2 measured by blood gas analysis (ABL 800Flex, Radiometer GmbH, Krefeld, Germany) with a target value between 400 and 500 mmHg [40]. Tissue oxygenation was measured with the FirestingPro (FSPRO-4, Pyroscience GmbH, Aachen, Germany) and the FirestingPro Sensor (OXIMP250, Pyroscience GmbH, Aachen, Germany). Further details are provided in the Supplementary Materials (Supplementary S4).

### 2.4. Perfusion Solution

Both groups were perfused with 2 L of Omnisol, supplemented with 120 mg dissolved creatinine (Sigma Aldrich Chemie GmbH, Taufkirchen, Germany) to enable the analysis of creatinine clearance. Furthermore, 750 mg cefuroxime (Dr. Friedrich Eberth Arzneimittel GmbH, Ursensollen, Germany), 16 mg dexamethasone (Fortecortin inject, Merck KgaA, Darmstadt, Germany) and 10,000 IU heparin (B. Braun Melsungen AG, Melsungen, Germany) were added to Omnisol before starting the perfusion. Insulin glulisine (Apidra, Sanofi-Aventis Deutschland GmbH, Frankfurt am Main, Germany) was continuously infused via syringe pumps at a rate of 33 IE/h.

### 2.5. Biochemistry

Samples were collected at the predefined time points, with first arterial perfusate samples taken at 0 min, first venous perfusate samples taken at 5 min and first urine samples taken at 30 min of perfusion. To collect urine samples, the ureter-catheter outlet was connected to a Falcon tube outside the wet chamber, and the time needed to accumulate 5 mL was measured to extrapolate urinary flow in mL/min. Afterwards, the catheter was placed back in the wet chamber to enable further urine recirculation, based on Weißenbacher et al. [41].

Blood gas analysis was performed with an in-line blood gas analyzer (ABL 800Flex, Radiometer GmbH, Krefeld, Germany), used for analyzing pH, partial pressure of carbon dioxide (pCO2), partial pressure of oxygen (pO2), electrolytes (potassium, sodium, calcium, chloride), glucose and lactate of arterial, venous and urine samples. pH, pO2 and pCO2 were temperature-corrected.

Arterial perfusate samples for subsequent analysis of aspartate aminotransferase (AST) activity, lactate dehydrogenase (LDH) activity, total protein concentration, urea concentration and creatinine concentration by the local DIN-ISO 9001:2015-certified laboratory [38], as well as urine samples to analyze urea concentration, urine protein concentration and creatinine concentration were stored at −80 °C after withdrawal. Intrarenal resistance (IRR) was calculated as $\frac{MAP}{renal\;flow}$, and the normalized creatinine clearance rate (CCr) was calculated as $\frac{urine\;creatinine\;\times \;urinary\;flow}{perfusate\;creatinine}÷kidney\;weight\times 100$. Fractional sodium excretion (FENa) was calculated as $\frac{\left(urine\;sodium/perfusate\;sodium\right)}{\left(urine\;creatinine/perfusate\;creatinine\right)}\times 100$ and fractional potassium excretion (FEK) as $\frac{\left(urine\;potassium/perfusate\;potassium\right)}{\left(urine\;creatinine/perfusate\;creatinine\right)}\times 100$.

### 2.6. Molecular Biomarkers

Core-needle biopsies were stored at −80 °C for Western blot (WB) analysis as previously described [38]. Tissue samples were assessed for expression of protein kinase B (AKT) and extracellular-signal-regulated kinase (ERK) as indicators for cell survival (high phosphorylated AKT (pAKT)/AKT ratio) and proliferation (high phosphorylated ERK (pERK)/ERK ratio). Proteins were separated via sodium dodecyl sulfate–polyacrylamide gel electrophoresis (SDS-PAGE). Total protein loading was monitored using stain-free technology on a ChemiDoc™ MP Imaging System (Bio-Rad). Proteins were transferred onto Polyvinylidenefluoride (PVDF) membranes using the Trans-Blot^®^ Turbo™ Transfer System (Bio-Rad Laboratories GmbH, Feldkirchen, Germany), and membranes were probed with specific primary and secondary antibodies as detailed in the Supplementary Materials (Supplementary S5). Protein signals were detected using ECL™ Prime (Sigma Aldrich Chemie GmbH, Taufkirchen, Germany) and quantified via Image Lab software (Version 6.1, Bio-Rad), with normalization to the total protein amount.

Analysis of Neutrophil Gelatinase-Associated Lipocalin (NGAL) (BioPorto Diagnostics, #KIT 036, Hellerup, Denmark) in urinary samples and Hypoxia Inducible Factor 1 Alpha (HIF-1α) (Elabscience, #E-EL-H6066, Wuhan, China) in perfusate samples was performed through enzyme-linked immunosorbent assays (ELISAs).

### 2.7. Statistical Analysis

For all statistical analyses, the GraphPad Prism 10.4.1 software package (GraphPad Software Inc., San Diego, CA, USA) was used. Perfusion parameters, blood-gas analysis, oxygenation, renal function and injury, including results from ELISAs, were tested for normal distribution using the Kolmogorov–Smirnov test, before a two-way analysis of variance (ANOVA) for multiple comparisons, followed by Šídák’s multiple comparisons test, was conducted. For the statistical analysis of WB results, a two-way ANOVA followed by uncorrected Fisher’s LSD multiple comparison was performed. Data are displayed as mean ± standard error of the mean (SEM), with *p*-values < 0.05 regarded as significant.

## 3. Results

For each analysis, a sample size of *n* = 6 per group was included.

### 3.1. Perfusion Parameters

**Figure 2:**
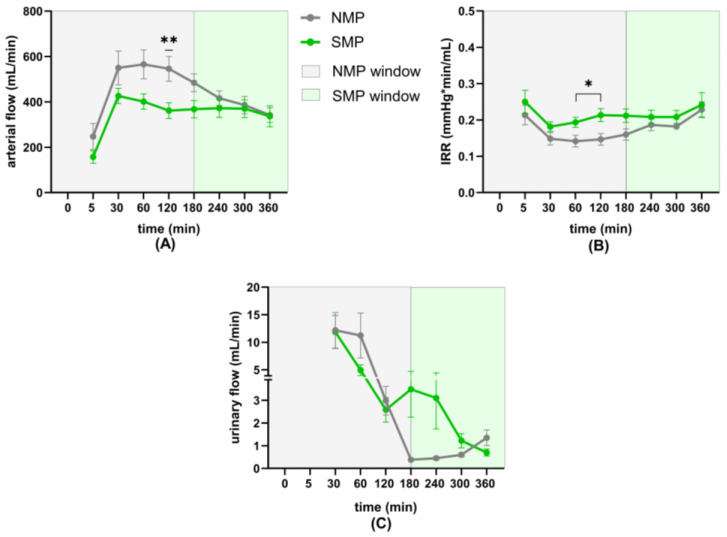
Renal perfusion parameters (**A**) arterial flow, (**B**) IRR, and (**C**) urinary flow. * *p* < 0.05; ** *p* < 0.01.

Arterial flow was consistently higher in the NMP group, reaching a significant difference at 2 h after a sharp increase from 5 to 30 min, consistent with the upregulation of MAP from 25 mmHg to 75 mmHg target pressure. After reaching its maximum at 1 h, arterial flow in the NMP group steadily decreased, but remained higher compared to the SMP group, which showed stable flow over 6 h perfusion (Figure 2A).

IRR was moderately higher in the SMP group and differed significantly at 1 h and 2 h (Figure 2B).

Although not significant, urinary flow was higher in the NMP group for the first 2 h, peaking at 30 min, but decreasing afterwards. The SMP group’s urinary flow also decreased over time, but was numerically higher than that of the NMP group between hours 2 and 5 (Figure 2C).

### 3.2. Renal Function

**Figure 3:**
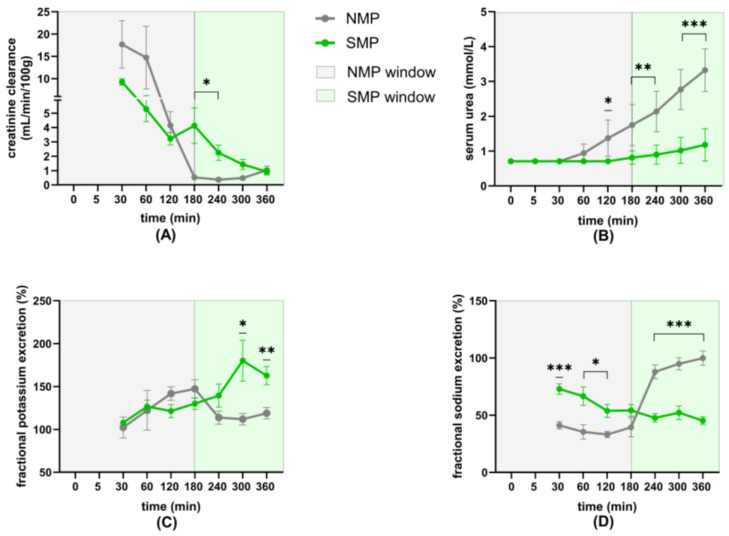
Renal function parameters (**A**) normalized CCr, (**B**) perfusate urea concentrations, (**C**) FEK, and (**D**) FENa. * *p* < 0.05; ** *p* < 0.01; *** *p* < 0.001.

At 3 and 4 h, CCr was higher in the SMP group compared to the NMP group (Figure 3A).

Perfusate urea concentration increased in both groups over time, but was significantly higher in the NMP group from 2 to 6 h (Figure 3B).

FEK was significantly higher in the SMP group at 5 and 6 h (Figure 3C). FENa in the NMP group was significantly lower during the first 3 h perfusion, followed by a rapid increase, in contrast to the SMP group, which showed a continuous decrease throughout 6 h perfusion, with significantly lower concentrations from 4 to 6 h (Figure 3D).

### 3.3. Perfusate Markers

**Figure 4:**
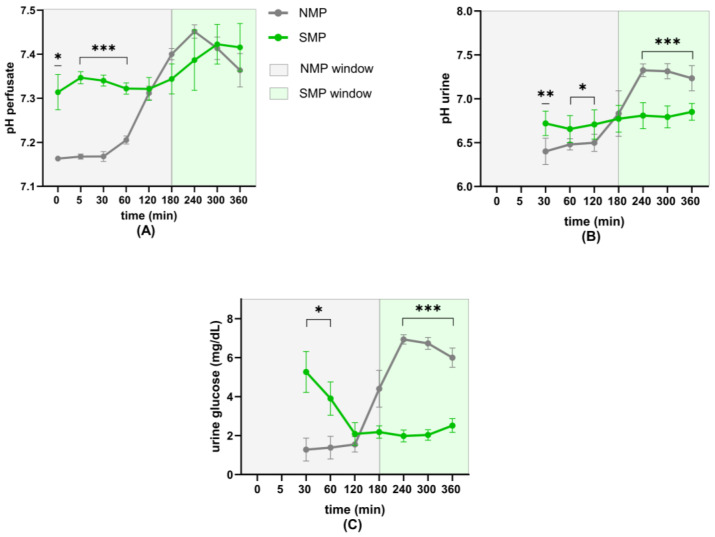
Perfusate and urine pH and urine glucose results: (**A**) Perfusate pH, (**B**) urinary pH, and (**C**) urinary glucose concentrations. * *p* < 0.05; ** *p* = 0.003; *** *p* < 0.001.

Perfusate pH remained stable in the SMP group, with comparable values at the start and end of perfusion. In contrast, the NMP group’s pH was significantly lower for the first hour, then increased to a maximum at 4 h, after which it decreased again (Figure 4A).

Urinary pH remained stable in the SMP group from the start to the end of perfusion. In contrast, the NMP group’s initial pH was significantly lower and remained lower up until 2 h, followed by an increase to significantly higher values compared to SMP for the remaining duration of perfusion (Figure 4B).

Urinary glucose concentrations were lower in the NMP group for the first 2 h of perfusion but increased afterwards and were significantly lower in the SMP group after 3 h until the end of perfusion (Figure 4C).

Electrolyte and glucose levels in perfusate and urine are displayed in Table 1. The NMP group had significantly higher arterial potassium concentrations after 6 h of perfusion compared to the SMP group. At 6 h, SMP kidneys showed significantly higher urinary potassium concentrations and lower urinary sodium concentrations than NMP kidneys. In the NMP group, urinary sodium and potassium concentrations were comparable to perfusate levels at 6 h. Figures displaying electrolyte changes over the total perfusion time are provided in the Supplementary Materials (Supplementary S6).

### 3.4. Oxygenation

Supraphysiological pO2 values were reached in both groups (total mean SMP: 434.60 ± 65.06 mmHg, NMP: 520.6 ± 30.22 mmHg). There was no significant difference between groups.

**Figure 5:**
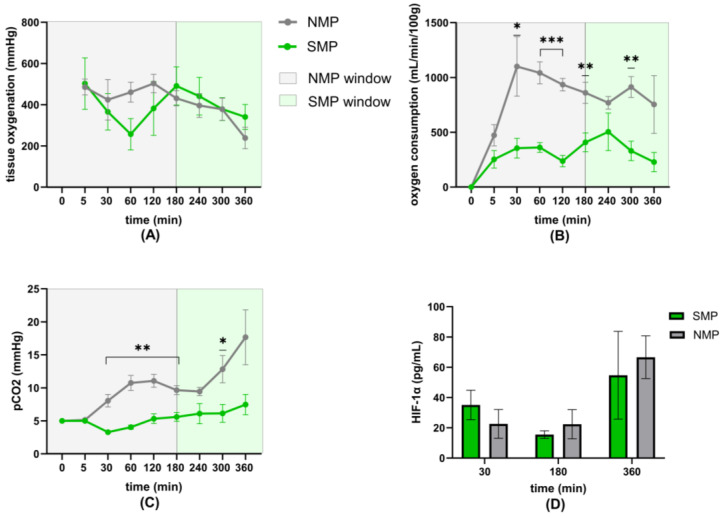
Oxygenation parameters: (**A**) Renal tissue oxygenation, (**B**) oxygen consumption per 100 g, (**C**) perfusate pCO2 concentrations, and (**D**) perfusate HIF-1α concentrations. * *p* < 0.05; ** *p* < 0.01; *** *p* < 0.001.

Throughout the entire perfusion, perfusate partial pressure of pCO2 was higher in the NMP group compared to the SMP group, with significant differences at timepoints 30 min–3 h and 5 h (Figure 5C).

Tissue oxygenation of the kidneys was achieved, with a mean of 393.5 ± 60.10 mmHg in the SMP and 401.8 ± 31.76 mmHg in the NMP group. Tissue oxygenation did not differ significantly between groups (Figure 5A).

Oxygen consumption was significantly higher in the NMP group throughout perfusion (Figure 5B).

HIF-1α concentrations in the perfusate remained low and did not show significant differences during perfusion, although there was a trend showing numerically higher levels of HIF-1α in SMP kidneys at 30 min, and higher levels in NMP kidneys at 3 and 6 h (Figure 5D).

### 3.5. Renal Injury

**Figure 6:**
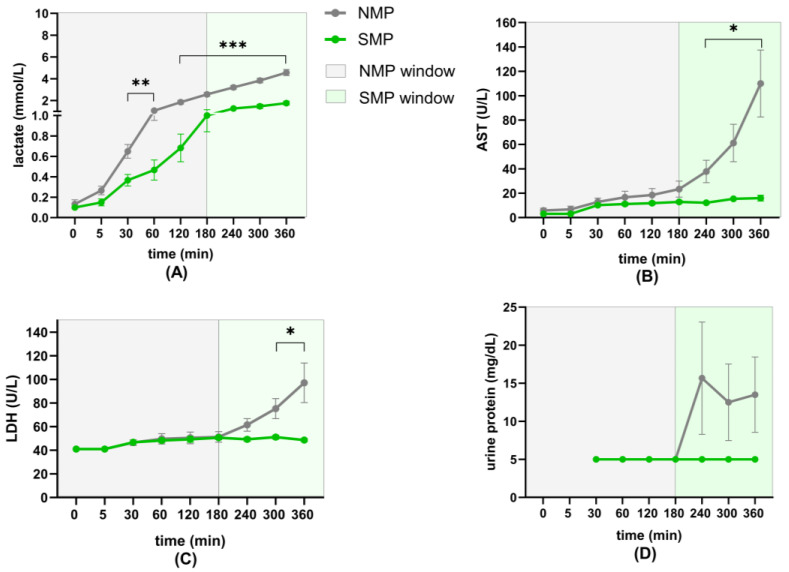
Markers of renal injury: (**A**) Perfusate lactate concentrations, (**B**) perfusate AST activity, (**C**) perfusate LDH activity, and (**D**) concentrations of urine protein (5 mg/dL cut-off). * *p* < 0.05; ** *p* < 0.01; *** *p* < 0.001.

Perfusate lactate levels were higher in the NMP group compared to the SMP group throughout perfusion. In both groups, an increase in lactate levels could be observed over time, with significant differences between 30 min and 1 h and between 2 and 6 h (Figure 6A).

Perfusate AST activity was higher in the NMP group throughout perfusion. Both groups showed an increase over time, but more extensively in the NMP group, especially after 3 h, leading to significant differences between the groups from 4 to 6 h in favor of SMP (Figure 6B).

Perfusate LDH activity did not differ significantly during the first 4 h of perfusion. However, at 5 and 6 h, LDH activity was significantly higher in the NMP group (Figure 6C).

Urine protein levels did not differ significantly during 6 h of perfusion due to high variation in the NMP group, but there was a clear trend of increasing urine protein amounts in the NMP group after 3 h, whereas levels in the SMP group remained consistently low (Figure 6D).

**Figure 7:**
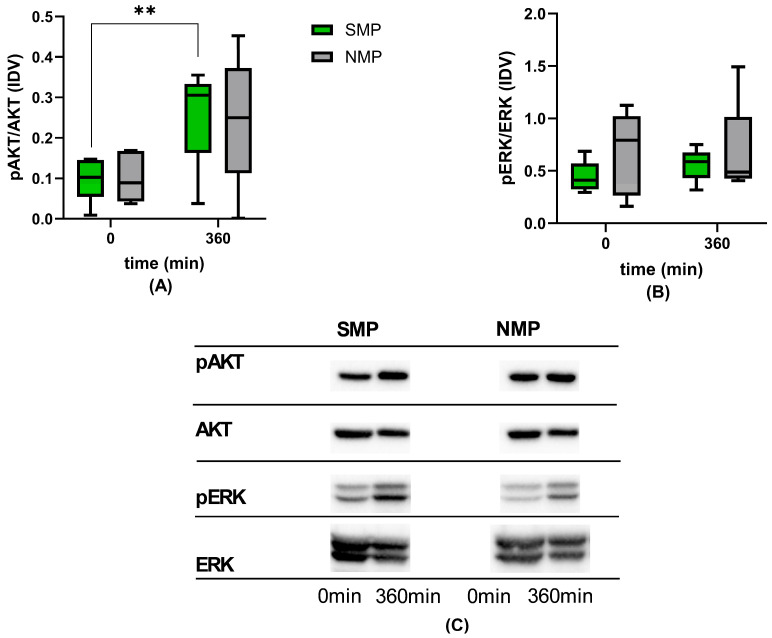
Cell survival and proliferation markers: (**A**) pAKT/AKT and (**B**) pERK/ERK. (**C**) Exemplary WB analysis at timepoints 0 and 6 h. ** *p* < 0.01.

The pAKT/AKT ratio was comparable for both groups and increased over time, with a significant increase in the SMP group from 0 min to 6 h of perfusion. The pERK/ERK ratio increased in both groups over time, showing no significant differences but indicated a trend of higher levels in NMP kidneys (Figure 7).

**Figure 8:**
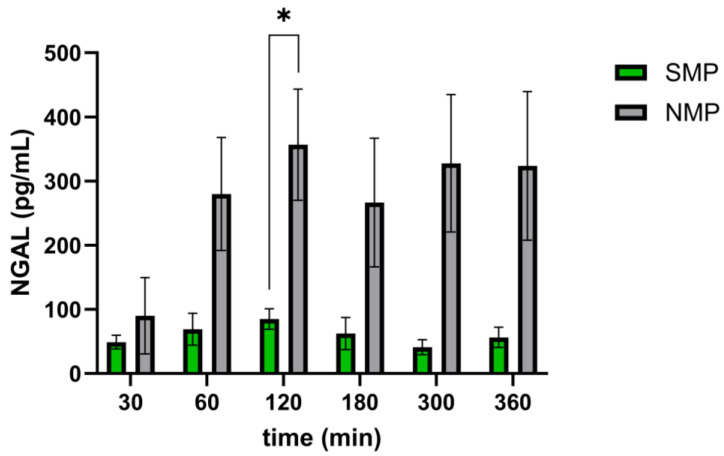
Urinary NGAL concentrations in SMP and NMP during 6 h of perfusion. * *p* < 0.05.

NGAL concentrations were higher in the NMP group, with a significant difference at 2 h (Figure 8).

## 4. Discussion

Improving organ preservation prior to transplantation is crucial for optimizing marginal graft utilization. Recent evidence indicates that ex vivo kidney perfusion at temperatures above hypothermia can improve preservation quality and the availability of transplantable organs [17,19,20,29].

Here, we compared ex vivo kidney perfusion with the novel fully synthetic perfusion solution Omnisol during 6 h in normothermic and subnormothermic perfusion. The major finding was that in this experimental setting with Omnisol, NMP appeared to be favorable during the initial 3 h of perfusion, whereas SMP was associated with overall stable and comparatively more favorable perfusion preservation during the 3–6 h window.

During the first 3 h of perfusion, NMP kidneys showed better renal function, as suggested by higher CCr, higher FEK, lower FENa and higher urinary flow. Additional indications of better function are lower urinary glucose concentrations and a urinary pH closer to the physiological value of 6.13 [42]. These findings suggest that NMP with Omnisol could be preferable when a 3 h functional assessment and/or treatment period is required. Beyond 3 h, renal function in SMP kidneys tended to be higher compared to kidneys in the NMP group. FEK increased, and FENa further decreased over the entire perfusion duration in SMP kidneys, suggesting potential advantages of SMP over NMP beyond 3 h. Furthermore, urinary flow remained in physiological ranges in SMP kidneys for the total perfusion period [43]. Perfusate urea levels increased over time in NMP kidneys, indicating a decrease in renal clearance, while remaining consistently low in SMP kidneys. Reduced function after 3 h is further indicated by similar electrolyte and glucose concentrations in perfusate and urine [44] in the NMP group at the end of perfusion, along with a decreasing perfusate pH after 4 h [45].

To simulate CCr during perfusion, we supplemented the Omnisol perfusate with creatinine [38]. Comparable creatinine concentrations in the perfusate at the beginning and end of perfusion (6.63 ± 1.14 mg/dL and 6.14 ± 0.54 mg/dL, respectively) indicate that creatinine remained at measurable concentrations in the recirculating system. CCr was comparable during the first 2 h, followed by a higher CCr in SMP at 3 and 4 h, which thereafter decreased in line with lower urine production, albeit still at physiological levels. This was corroborated by urine protein levels below the measuring threshold in the first 3 h in both groups [46], which persisted until 6 h in the SMP group, but increased in the NMP group after 3 h. Tubular function, as expressed by FENa and FEK, indicated a benefit of NMP in the first 3 h, and SMP surpassed NMP after 3 h. An increase in urinary glucose concentrations after 3 h in NMP kidneys seems to confirm these findings [47]. Moreover, the significant increase in the pAKT/AKT ratio in the SMP group indicates activated cell survival pathways beyond the 3 h NMP window [48].

In terms of injury markers, AST and LDH represent non-organ-specific indicators of cellular membrane disruption and necrosis, associated with cumulative parenchymal injury during perfusion. In isolated organ perfusion settings, as in the present study, increased perfusate activity of AST and LDH originates exclusively from renal tissue and therefore directly indicates kidney injury. NGAL, a well-established early biomarker of tubular injury, indicates stress predominantly at the tubular epithelial level [49,50]. Glomerular filtration capacity and barrier integrity can be evaluated via the measurement of urine protein concentrations, whereas lactate serves as a sensitive marker, especially regarding insufficient oxygen utilization. In the present study, NMP kidneys exhibited numerically higher levels of these markers over time, consistent with active metabolism and ongoing cellular turnover under normothermic conditions. However, both absolute concentrations and delta changes remained lower compared to blood-based NMP protocols [38,39,51], suggesting reduced confounding by hemolysis-associated enzyme release and indicating a potential cytoprotective effect of the acellular perfusate used in this experimental study. Despite being oxygen-carrier-free, the perfusate seemed to have met the oxygen requirements of the kidneys under both subnormothermic and normothermic conditions, as HIF-1α levels did not increase significantly in both groups between the start and end of perfusion. HIF-1α served as a marker for hypoxic conditions due to its central role in regulating downstream cellular processes under hypoxia [52]. Lower oxygen consumption in SMP kidneys is in accordance with lower metabolic demands.

Previous studies have demonstrated that blood-based NMP enables kidney resuscitation and functional assessment under physiological conditions [17,19,20]. Our findings suggest that similar processes may also be achievable using a fully synthetic perfusate, not only during NMP but potentially also with SMP. Our group has extensive experience with blood-based perfusates under NMP conditions [38,39,53,54], following established protocols, enabling reliable comparative analyses with cell-free solutions and varying temperature regimens. In relation to the these previously published datasets using blood based warm perfusion, the recent dataset using fully synthetic perfusion, shows 10–25 times lower urine protein levels, 4–5 times lower LDH, 2–3 times lower AST, 1.5 times lower lactate concentration in the perfusate, whilst functional parameters, like the creatinine clearance peaked at the same time at around 1 h of NMP, and fractional sodium and potassium excretion were non inferior in comparison between blood and fully synthetic NMP.

While blood-based perfusion remains the standard in NMP, its application in subnormothermic settings is limited by increased blood viscosity, erythrocyte aggregation, and impaired microcirculatory flow, potentially compromising oxygen delivery and endothelial integrity [55]. Although beneficial effects of blood-based subnormothermic perfusion have been reported in porcine kidneys with adjunctive strategies [35], cell-free perfusates offer improved rheological stability and may therefore be preferable [14]. Therefore, adding a group of SMP–blood-based perfusion to the recent setting of SMP–fully synthetic perfusion would have been contraindicatory to the physiological properties of naive blood-based perfusion. However, in future investigations, we aim to validate the SMP and NMP findings. Accordingly, future large-animal studies will include a porcine kidney autotransplantation model, enabling assessment of post-transplant graft function and injury after preservation with Omnisol. Furthermore, evaluation of clinically relevant functional endpoints will be important to further explore the translational potential of this synthetic perfusate. Despite the validation from large-animal studies, investigations on human kidneys are required to be able to translate our findings to a clinical setting.

Concerning autotransplantation models, Abraham et al. compared 4 h of SMP to SCS using porcine kidneys, followed by autotransplantation, and showed better graft function with SMP [36]. However, their protocol used a human albumin-based perfusate, which is cost-intensive [56] and limited by the general shortage of human blood plasma, making it unsuitable for routine clinical application [57]. Agius et al. demonstrated superior post transplantation graft metabolism and reduced IRI after SMP compared to SCS in a porcine model, although the study was limited to 4 h of perfusion and 1 h post-transplant follow-up [34].

Thus, when comparing our recent protocol for a potential application in porcine autotransplantation models to the ex vivo perfusion requirements of an established clinical setting for kidney resuscitation, including a 90 min controlled oxygenated rewarming phase and the assessment of vital parameters after 2 h of perfusion at 35 °C, as published by Jaynes and colleagues [58], our protocol meets both the experimental and clinical requirements of the Sub-Normothermic Acellular Perfusion (SNAP) protocol. Additionally, Omnisol does not require human albumin as a rare and expensive colloid but instead uses polyethylene glycol as the colloid. Furthermore, it enables NMP as a technology that is increasingly recognized as an accepted organ preservation and assessment tool, as recently published by Holzner and colleagues [59].

Future perspectives of SMP and NMP warrant further exploration, as each method offers unique and complementary advantages for organ preservation.

To finally confirm translational significance, studies on SMP for human kidneys are required. De Haan et al. already demonstrated that SMP enables up to 4 days of perfusion in discarded human kidneys; however, they used a human albumin-based perfusate [29].

The authors are aware of several limitations within this ex vivo study. Firstly, larger or older animals, or animals from different species, might also have influenced the study results, as De Haan and colleagues recently published evidence that porcine kidneys do not reflect human kidney perfusion dynamics, at least due to different endothelial cell integrity, leading more often to swelling and malfunction [29]. Secondly, within this experimental setup, the perfusion pressure was adapted with an arterial pressure of 75 mmHg. We used a centrifugal continuous flow pump, not a roller pump. A lower or higher-pressure setup would have had an immediate effect on the flow rate and is therefore a major risk of bias. We also used 100% medical oxygen for the oxygenation of the perfusion circuit. The use of Carbogen (95% Oxygen/5% CO_2_) may also influence pH. Additionally, the relatively small number of kidneys per group, which reflects the ethical commitment to the 3R principle, however, limits the statistical power and generalizability of our findings. Furthermore, the absence of a blood-based perfusion control group, also reflecting commitment to the 3R principle, is a limitation of this study. Nonetheless, previous comparable studies can serve as a reference for the interpretation of our study [38,39,53,54].

## 5. Conclusions

Both NMP and SMP offer distinct advantages for kidney preservation, and the synthetic perfusate Omnisol appears to be feasible for both methods. In this experimental setting, the data indicate that NMP of porcine kidneys was associated with favorable functional parameters during the early phase of perfusion, whereas SMP showed comparatively stable parameters at later time points. These findings should primarily be considered exploratory observations and require validation in future studies, especially for the translation into a clinical scenario using human kidneys.

## Figures and Tables

**Table 1:** Concentrations of electrolytes and glucose at the beginning and after 6 h of perfusion.

Arterial Perfusate	SMP 5 min	NMP 5 min	SMP 360 min	NMP 360 min
K^+^ (mmol/L)	10.53 ± 0.10	10.48 ± 0.26	9.6 ± 0.15	10.52 ± 0.1 (***)
Na^+^ (mmol/L)	104.6 ± 0.4	104.2 ± 1.7	109.3 ± 0.84	113.2 ± 1.0 (*)
Ca^2+^ (mmol/L)	0.28 ± 0.01	0.29 ± 0.0	0.29 ± 0.01	0.3 ± 0.01
Cl^−^ (mmol/L)	35.2 ± 0.37	37.33 ± 1.38	39.17 ± 0.95	42.5 ± 0.96 (*)
Glucose (mg/dL)	4.52 ± 0.07	4.600 ± 0.07	6.97 ± 0.23	6.47 ± 0.37
urine	SMP 30 min	NMP 30 min	SMP 360 min	NMP 360 min
K^+^ (mmol/L)	14.3 ± 1.01	17.63 ± 1.71	25.0 ± 0.0	12.07 ± 1.01 (***)
Na^+^ (mmol/L)	93.17 ± 2.15	83.33 ± 1.5 (**)	74.5 ± 5.12	107.5 ± 3.7 (***)
Ca^2+^ (mmol/L)	0.25 ± 0.01	0.22 ± 0.01 (**)	0.25 ± 0.01	0.28 ± 0.01
Cl^−^ (mmol/L)	18.83 ± 3.09	7.0 ± 0.0 (*)	11.83 ± 1.94	40.0 ± 1.29 (***)
Glucose (mg/dL)	5.27 ± 1.05	1.28 ± 0.59 (*)	2.52 ± 0.35	6.0 ± 0.49 (***)

Significance markers refer to the comparison of SMP vs. NMP at specific time points. * *p* < 0.05; ** *p* < 0.01; *** *p* < 0.001.

## Data Availability

The data supporting the conclusions of this article will be made available by the authors on request.

## Supplementary Materials

The following supporting information can be downloaded at: https://www.mdpi.com/article/10.3390/jcm15062287/s1, Additional information on surgical procedures, materials for sample collection, perfusion circuit details, tissue oxygenation measurement and molecular biomarkers; Suppl.1: Additional information on surgical procedure; Suppl.2: Additional information on materials used for sample collection; Suppl.3: Additional information on perfusion circuit details; Suppl.4: Additional information on tissue oxygenation measurement; Suppl.5: Additional information on molecular biomarkers; Suppl. 6: Figures of electrolytes.

## Abbreviations

The following abbreviations are used in this manuscript:

AKT	Protein kinase B
ANOVA	Analysis of variance
AST	Aspartate aminotransferase
CCr	Creatinine clearance
DCD	Donation after circulatory death
DGF	Delayed graft function
ECD	Extended criteria donor
ELISA	Enzyme-linked immunosorbent assay
ERK	Extracellular-signal-regulated kinase
FEK	Fractional potassium excretion
FENa	Fractional sodium excretion
h	Hours
HBOCs	Hemoglobin-based oxygen carriers
HIF-1α	Hypoxia inducible factor 1 alpha
HOPE	Hypothermic oxygenated perfusion
IRI	Ischemia–reperfusion injury
IRR	Intrarenal resistance
LDH	Lactate dehydrogenase
MAP	Mean arterial pressure
min	Minutes
NGAL	Neutrophil gelatinase-associated lipocalin
NMP	Normothermic machine perfusion
pAKT	Phosphorylated protein kinase B
pCO_2_	Partial pressure of carbon dioxide
pERK	Phosphorylated extracellular-signal-regulated kinase
PNF	Primary non-function
pO_2_	Partial pressure of Oxygen
PVDF	Polyvinylidenefluoride
RBCs	Red blood cells
SCS	Static cold storage
SDS-PAGE	Sodium dodecyl sulfate–polyacrylamide gel electrophoresis
SEM	Standard error of the mean
SMP	Subnormothermic machine perfusion
WB	Western blot
