# Supplementary material for: Comparison of Normothermic and Subnormothermic Machine Perfusion of Porcine Kidneys Using a Novel Fully Synthetic Perfusion Solution: A Proof-of-Concept Study
Source: J Clin Med. 2026 Mar 17;15(6):2287. doi: 10.3390/jcm15062287 (PMC13027045; doi:10.3390/jcm15062287)
Supplement: Supplementary file 1 [file jcm-15-02287-s001.zip › jcm-4152373-supplementary.pdf]

Supplementary file

Suppl.1: Additional information on surgical procedure:

After premedication animals were intubated and volume-controlled mechanical normoventilation was performed maintaining normocapnia. Continuous monitoring of heart rate, oxygen saturation and ECG was executed. The animals received Isoflurane (end-tidal concentration of 1-1.5 vol. %), and fentanyl (10 µg/kg BW/h) was administered for analgesia. After an adequate depth of narcosis, the animals underwent a laparotomy, and the kidneys and other organs (e.g. liver, heart, lungs etc.) were explanted and used for various ex-vivo experiments. Euthanasia was performed by an overdose of intravenous (i.v.) application of pentobarbital (80 mg/kg BW) (Narcoren, Merial GmbH, Hallbergmoss, Germany) and exsanguination under deep anaesthesia

Suppl.2: Additional information on materials used for sample collection

Besides the cannulation of the artery, the renal vein (Thomafluid® Luer Lock Tubing Adapter Female) together with a flexible tube, ID 6.4 mm (1/4”), Reichelt Chemietechnik GmbH + Co., Heidelberg, Germany) and ureter (Suction Catheter ProFlo straight tip with a funnel, 14 Ch., ConvaTec GmbH, Munich, Germany) were cannulated for sample collection.

Suppl.3: Additional information on perfusion circuit details

Kidneys were placed in the wet chamber and connected to the circuit via the arterial catheter. The urinary catheter was placed in the container, allowing continuous urinary recirculation and punctual urine collection at sampling time points. Perfusate was draining freely from the canulated renal vein. The circuit included a reservoir (Trilly Pediatric AF, Eurosets GmbH, Gröbenzell, Germany), a centrifugal blood pump (Affinity CP, Medtronic GmbH, Meerbusch, Germany), an oxygenator (Newborn A.L.ONE ECMO, Eurosets GmbH, Gröbenzell, Germany), and a paediatric arterial filter (AffinityTM, Medtronic GmbH, Meerbusch, Germany). A custom-designed pump controller (Informatik 11-Embedded Software, RWTH Aachen University, Aachen, Germany) was used to operate the centrifugal blood pump with a control unit for RPM.

An ultrasonic flow meter (SonoTT, em-tec GmbH, Finning, Germany) recorded the arterial renal perfusate flow (RBF), and MAP and temperature were continuously measured (IntelliVue MX500, Royal Philips Electronics, Amsterdam, The Netherlands).

RBF, pump RPM, MAP, temperature, urinary flow and tissue oxygenation were recorded at each predefined time point.

Suppl.4: Additional information on tissue oxygenation measurement

The Firesting sensor was inserted into the renal cortex through the stich incision formed by the core needle biopsy prior to perfusion. Measurement values were automatically temperature corrected via the Firesting temperature sensor (TDIP15, Pyroscience GmbH, Aachen, Germany), placed underneath the kidney.

Suppl.5: Additional information on molecular biomarkers

WB analysis: The respective primary antibodies (pErk1/2: #9101; Erk1/2: #9102; pAkt: #9271; Akt: #4691; Cell Signaling Technology, Leiden, The Netherlands; Vinculin: V9131, Sigma-Aldrich Chemie GmbH, Taufkirchen, Germany) and secondary antibodies (anti-rabbit: #7074, Cell Signaling Technology, Leiden, The Netherlands; anti-mouse: GENXA9311ML, Sigma-Aldrich Chemie GmbH, Taufkirchen, Germany) were applied.

ELISA: For the HIF-1α ELISA, samples were undiluted. Of samples for NGAL ELISA, all SNMP samples and the 30minutes NMP samples were diluted 1:20, and the remaining NMP samples 1:200.

Suppl. 6: Figures of electrolytes

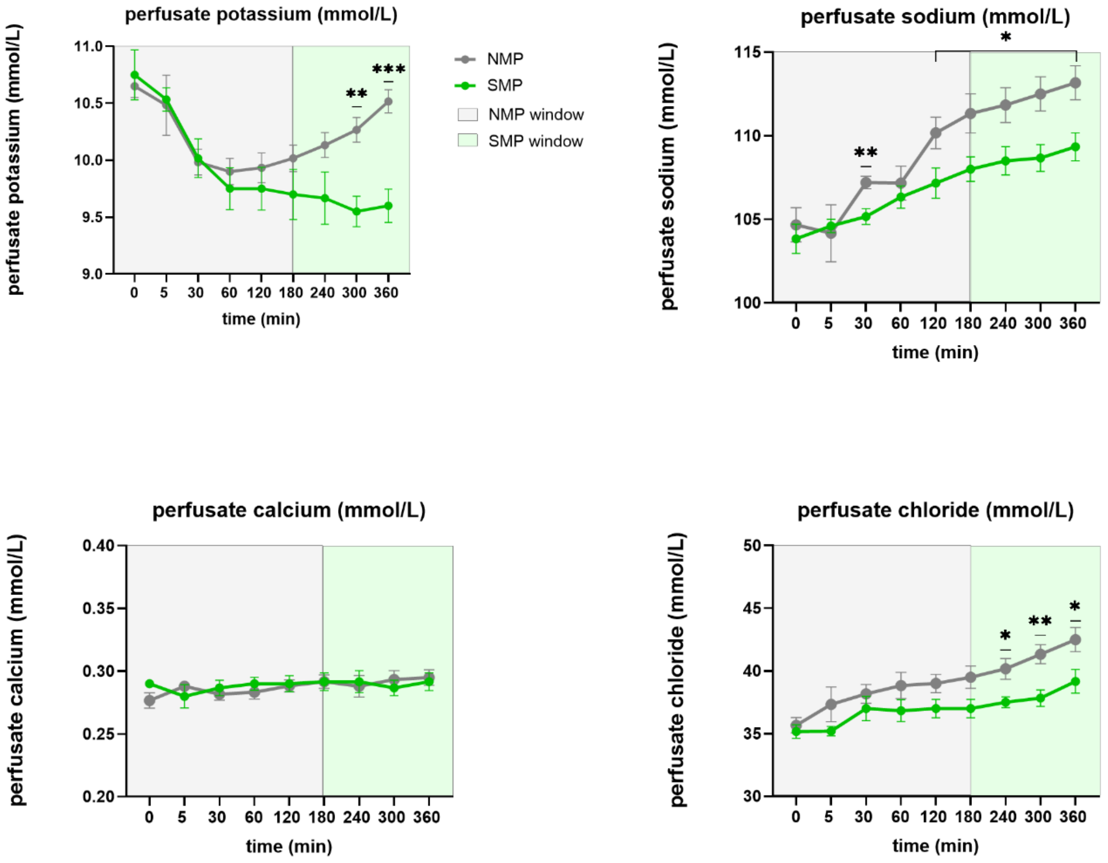

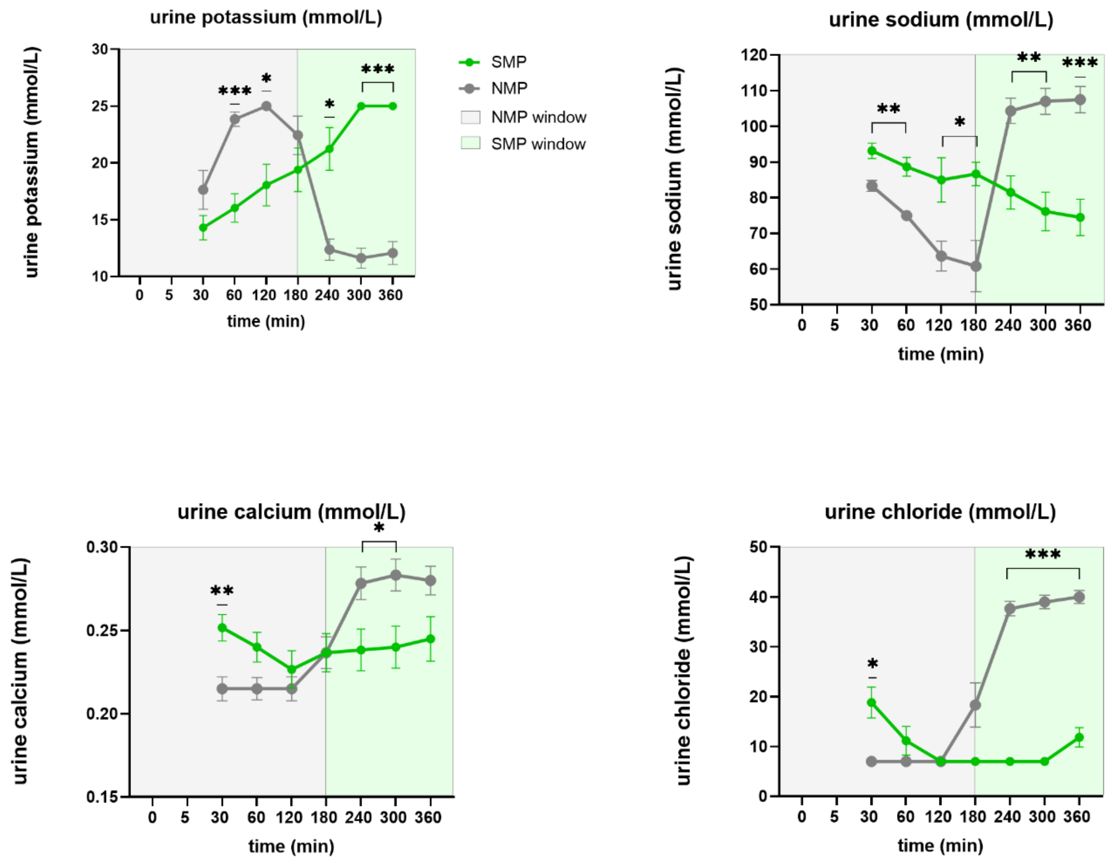

Note: Maximum of potassium detectable by blood gas analyser was 25 mmol/L.
